# The hypoxia-driven crosstalk between tumor and tumor-associated macrophages: mechanisms and clinical treatment strategies

**DOI:** 10.1186/s12943-022-01645-2

**Published:** 2022-09-08

**Authors:** Ruixue Bai, Yunong Li, Lingyan Jian, Yuehui Yang, Lin Zhao, Minjie Wei

**Affiliations:** 1 Department of Pharmacology, School of Pharmacy, China Medical University, Shenyang, 110122 People’s Republic of China; 2 Liaoning Key Laboratory of Molecular Targeted Anti-Tumor Drug Development and Evaluation, China Medical University, Shenyang, 110122 People’s Republic of China; 3 Department of Pharmacy, Shengjing Hospital of China Medical University, Shenyang, 110004 People’s Republic of China; 4 Shenyang Kangwei Medical Laboratory Analysis Co. LTD, Shenyang, 110000 People’s Republic of China

**Keywords:** Hypoxia-inducible factor, Oxygen sensor, Intercellular communication, Inhibitor

## Abstract

Given that hypoxia is a persistent physiological feature of many different solid tumors and a key driver for cancer malignancy, it is thought to be a major target in cancer treatment recently. Tumor-associated macrophages (TAMs) are the most abundant immune cells in the tumor microenvironment (TME), which have a large impact on tumor development and immunotherapy. TAMs massively accumulate within hypoxic tumor regions. TAMs and hypoxia represent a deadly combination because hypoxia has been suggested to induce a pro-tumorigenic macrophage phenotype. Hypoxia not only directly affects macrophage polarization, but it also has an indirect effect by altering the communication between tumor cells and macrophages. For example, hypoxia can influence the expression of chemokines and exosomes, both of which have profound impacts on the recipient cells. Recently, it has been demonstrated that the intricate interaction between cancer cells and TAMs in the hypoxic TME is relevant to poor prognosis and increased tumor malignancy. However, there are no comprehensive literature reviews on the molecular mechanisms underlying the hypoxia-mediated communication between tumor cells and TAMs. Therefore, this review has the aim to collect all recently available data on this topic and provide insights for developing novel therapeutic strategies for reducing the effects of hypoxia.

## Introduction

**Fig. 1:**
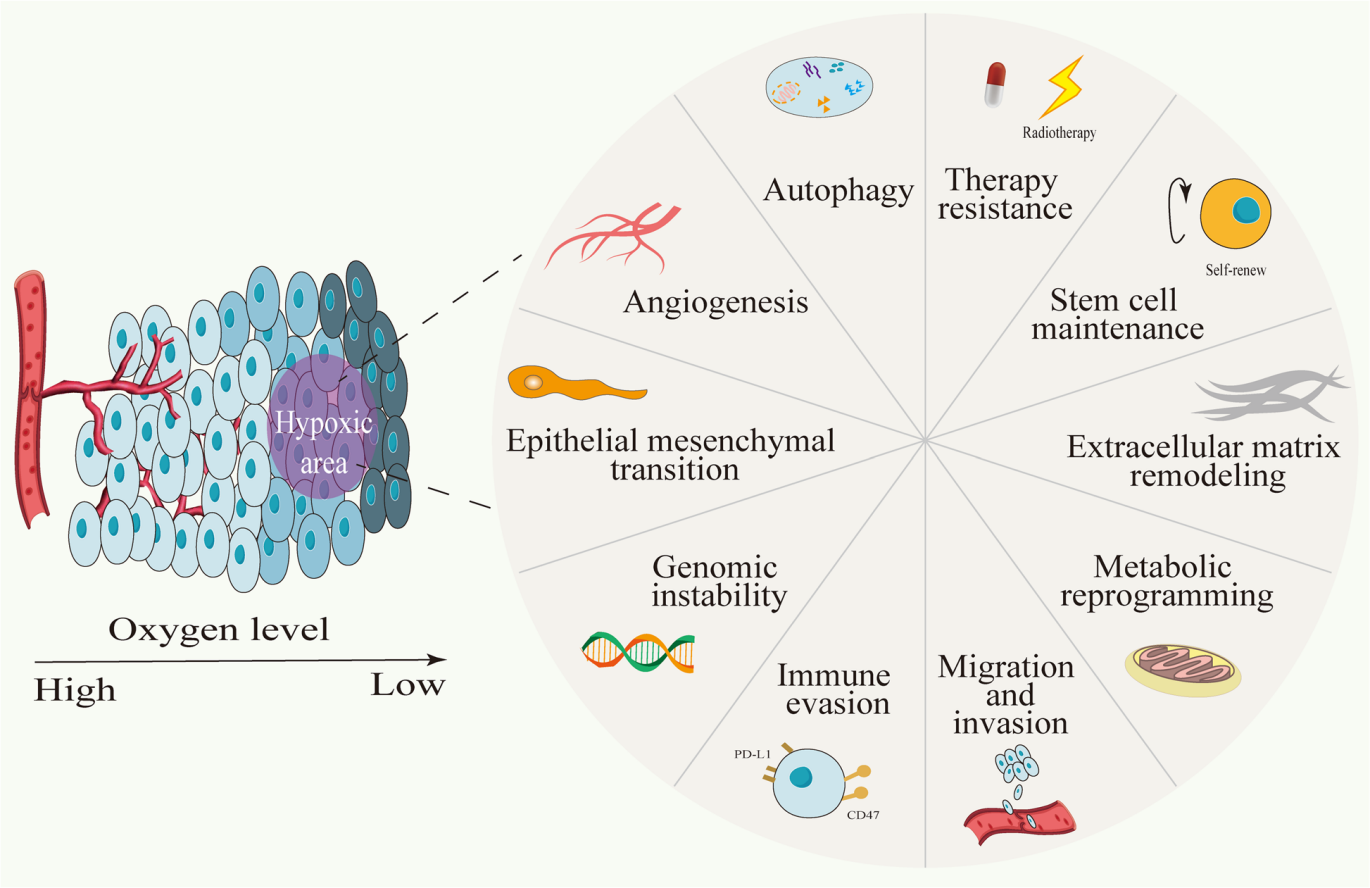
Overview of the effects of hypoxia on tumor cells. Hypoxia contributes to many critical aspects of cancer, including genome instability, autophagy, metabolic reprogramming, angiogenesis, migration, invasion, extracellular matrix remodeling, epithelial mesenchymal transition (EMT), stem cell maintenance, immune evasion and therapy resistance

As a prominent feature of solid tumors, hypoxia is thought to be a common cause of poor patient prognosis and therapeutic outcomes [1–3]. There is an increasing number of hypoxia-related publications highlighting its importance in tumors [4]. Studies have shown that long-term hypoxia is the main driving force of cancer development [5, 6]. According to the in vitro and tumor xenograft studies [7], even minutes of exposure of tumor cells to ambient air is enough to induce signaling alterations that affect their biology. Most preclinical studies collect and process tumor tissues in normoxia rather than physioxia, which contributes to therapy failure in clinic despite promising preclinical results [7]. Hypoxia contributes to various critical aspects of cancer, including genome instability [8], autophagy [9], metabolic reprogramming [10, 11], angiogenesis [12], migration, invasion [13], extracellular matrix remodeling [14], epithelial mesenchymal transition (EMT) [15], stem cell maintenance [16], immune evasion [17] and therapy resistance [18] (Fig. 1). Furthermore, in response to hypoxic stress, intercellular communication becomes more frequent and complex [19]. Therefore, hypoxia is thought to be a big obstacle to overcome in the treatment of malignancies [20].

Increasing evidence supports the vital role of TME during cancer development [21]. Complex TME is composed of various cells, including stromal cells and immune cells. Macrophages are centered in the innate immune system [22] and can acquire distinct functional properties in response to environmental stimuli [23]. A macrophage spectrum is a popular model for describing the properties of macrophage activation, with M1 and M2 macrophages at opposite ends and other types of macrophages in between [23, 24]. Within the cancerous tissue, macrophages can be referred as TAMs, that involve in every part of the tumorigenesis [22, 25, 26]. M1-like macrophages have tumor-suppressing properties, whereas M2-like macrophages have tumor-promoting properties [27]. Thus, TAMs are important regulators of tumor immunity and immunotherapy [28]. Recent literature suggested that higher proximity of tumor cells to M2-like TAMs correlates to lower overall survival rates [29]. Notably, hypoxia can drive macrophages to polarize into immune-suppressive [28] or angiogenic phenotypes [30]. For example, hypoxia directly drives macrophage polarization through the unfolded protein response pathways [31]. TAMs are preferentially presented in the hypoxic region [29, 32, 33]. The attraction of macrophages by various chemoattractants and the hampered mobility of macrophages in the hypoxic region are plausible mechanisms accounting for the accumulation of TAMs within the hypoxic niche [34, 35]. Once entrapped, macrophages will gradually polarize into M2 subtypes and serve protumoral functions [22]. Together with intra-tumor hypoxia, M2 phenotype TAMs can drive tumor aggressiveness [36] and severely restrict to the efficacy of immunotherapy [37]. Thus, in order to better understand the functional roles of TAMs in tumor progression, the effects of hypoxia should be taken into account.

It is well known that the crosstalk between TAMs and tumor cells plays a fundamental role in driving cancer progression [38]. The interaction of cancer cells with TAMs in the hypoxic TME, in particular, plays a significant role in tumorigenesis and may be a novel therapeutic target in cancer [34]. According to the most recent single-cell RNA-Seq data, hypoxia is the most important factor influencing cell communication [39]. Chemokines and exosomes are both crucial mediators of the crosstalk between TAMs and tumor cells. Recent in vivo and in vitro studies have shown that hypoxia can alter the secretion of chemokines and exosomes [40, 41]. The attraction of macrophages to tumor cells can be increased by exposing tumor cells to hypoxia [42]. In addition to the direct effects of hypoxia on macrophage polarization, hypoxia can also indirectly affect this process by altering the communication of tumor cells with macrophages [43]. Interestingly, some studies showed that blocking the CD47-SIRPα “don’t eat me signal” to promote macrophage phagocytosis of cancer cells may be ineffective in hypoxic colorectal cancer [44]. Despite the fact that the importance of hypoxia in oncology is now widely recognized, understanding the many complex interactions of hypoxia and related TME stresses with cancer biology and therapy remains a work in progress [45]. Additionally, there are some excellent comprehensive reviews on the interplay between tumor cells and macrophages [46–49], but there are few reviews on the crosstalk mediated by hypoxia. Therefore, in order to identify new therapeutic targets, it is urgently necessary to have a thorough understanding of the intricate mechanisms underlying the hypoxia-mediated interaction between TAMs and tumor cells.

In this review, we provided a piece of detailed information on pathophysiological features of tumor hypoxia and its mechanism in terms of sensing oxygen. Besides, we also summarized recent results in the experiments that focused on hypoxia-driven interaction between TAMs and tumor cells. Lastly, we analyzed current clinical strategies for limiting hypoxia-induced responses.

## Hypoxic tumor microenvironment

### Pathophysiologic features of tumor hypoxia

The majority of solid tumors, like normal tissues, require effective clearance of produced cellular metabolic wastes in addition to regular oxygen and nutrient supplies. The host blood vessels surrounding tumors are unable to meet the above demands of tumors due to the rapid proliferation of tumor cells. To compensate, tumors generate their own vasculature. Unfortunately, the tumor neo-vasculature is abnormal in structure and function. This situation makes the hostile tumor microenvironment to be characterized by poor perfusion, insufficient oxygen, nutritional deprivation, low pH, and elevated interstitial fluid pressure [50]. The oxygen level of tumor tissue lower than 10 mmHg (1.3 kPa) is defined as hypoxia [51].

Generally, tumor tissues harbor three regions in terms of different oxygen levels: normoxic region (with functional blood vessels nearby), hypoxic region (100 μm away from functional blood vessels), and necrotic region (150 μm away from functional blood vessels) [19]. Due to different mechanisms and duration of hypoxia, tumor hypoxia can be roughly divided into chronic hypoxia and acute hypoxia. Within each class, it can be further categorized into different subtypes according to the involved pathogenetic processes [50, 52–55]. Chronic hypoxia is caused primarily by diffusion limitations due to increased diffusion distances and adverse diffusion geometries. Uncontrolled development of tumors can cause some tumor cells located far away from blood vessels and thus be deprived from sufficient oxygen. A small proportion of chronic hypoxia is attributed to hypoxemia (e.g. the formation of HbCO in anemic patients and heavy smokers) and compromised perfusion of microvessels (e.g. disturbed starling force or solid-phase stress in tumor). On the other hand, acute hypoxia, also known as cyclic, intermittent, transient, repetitive, or fluctuating hypoxia, is primarily caused by temporal flow blocking in microvessels (e.g. blockage of blood vessels by cell aggregates and fibrin plugs) and transient hypoxemia (e.g. fluctuating red blood cell fluxes).

Of note, other studies have described three types of hypoxia: chronic hypoxia, acute hypoxia, and cyclic hypoxia [56]. The first type of hypoxia—chronic hypoxia is caused by over-proliferation of cancer cells with a key character of prolonged timescales (> 24 h). The second type of hypoxia—acute hypoxia arises is resulting from sudden blockages of small blood vessels, and it may last from few minutes to few hours (< 24 h). The third type of hypoxia—cyclic hypoxia (also referred as intermittent hypoxia or IH) is due to the short-term shutdown of immature tumor vasculature ranging from several minutes to days, which can be reversed by restoring blood flow [56, 57]. In this classification, cyclic hypoxia is characterized by the presence of cycles of hypoxia and reoxygenation (H-R cycles), whereas acute hypoxia is not followed by reoxygenation.

The total duration of hypoxia, oxygen concentration, and frequency of H-R cycles are three indicators that have a significant impact on the regulation of molecular mechanisms. Oxygen levels are believed to be correlated with tumor types [4, 58, 59]. There is no unambiguous and uniform classification system. One of the possible explanations is that there is currently no agreement on the methods for studying tumor hypoxia in vitro or in vivo [56, 60]. Therefore, there is an urgent need to standardize methods to recreate intratumoral hypoxia in the laboratory and detect intratumoral hypoxia in the clinic. A variety of hypoxia-mimicking model systems and technologies for quantification of hypoxia levels emerge as time requires [1, 61–63], which is expected to help us gain deeper insights in pathophysiological hallmarks of tumors and the mechanisms for adaptation to hypoxia.

### Oxygen sensing mechanisms

The oxygen level in hypoxic tumor region dynamically changes as tumor progression [64], thus understanding the molecular mechanism by which cells dominate the oxygen regulation is of great significance for cancer treatment. Drs. William G. Kaelin, Jr., Peter Ratcliffe,and Gregg Semenza won the 2019 Nobel Prize in Physiology or Medicine for their outstanding discoveries of cellular oxygen-sensing mechanisms.

**Fig. 2:**
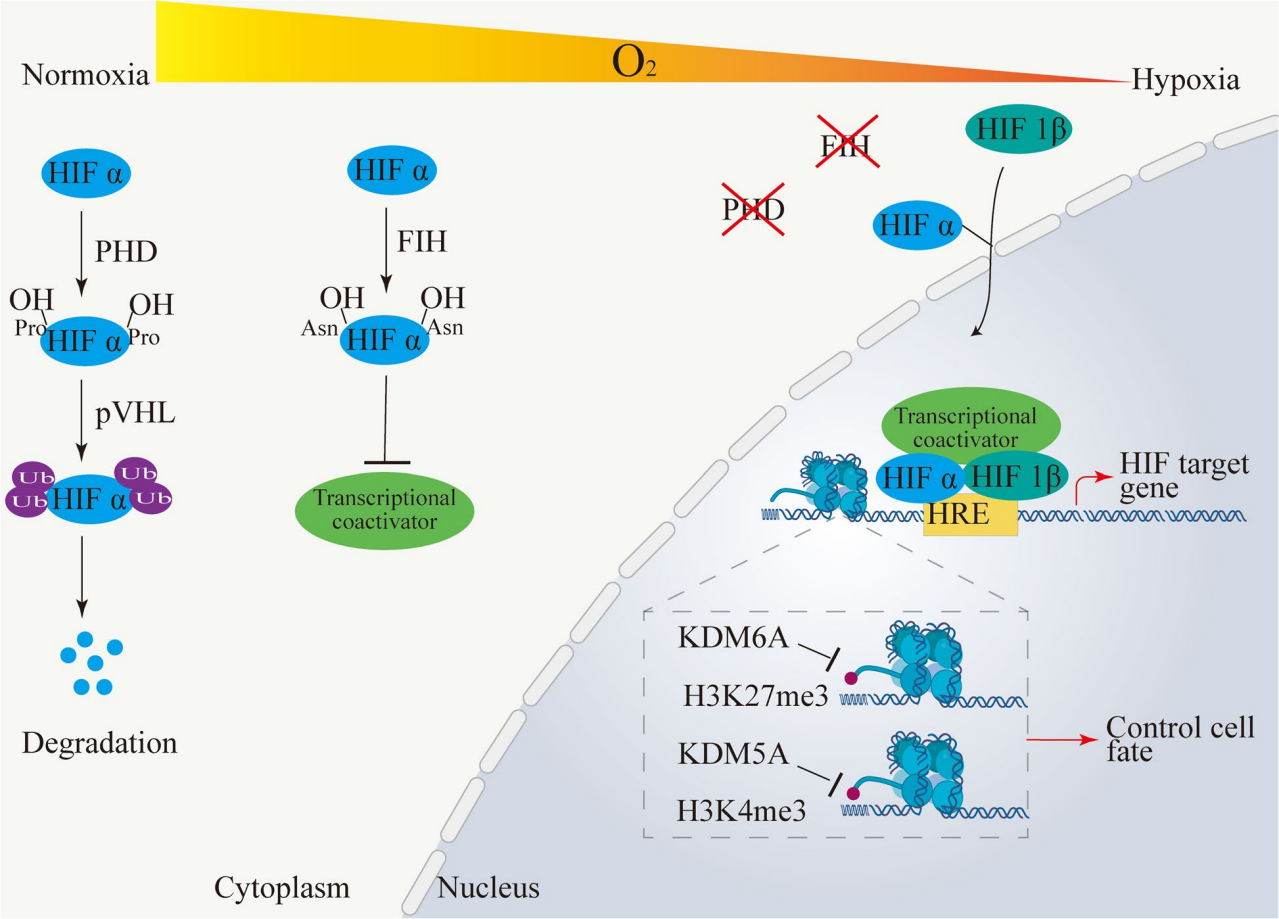
Oxygen sensing mechanisms. In presence of oxygen, HIF α is hydroxylated by prolyl hydroxylase (PHD) and FIH (factor inhibiting HIF), leading to rapid proteosomal degradation mediated by von Hippel–Lindau (VHL) protein and failure of recruiting transcriptional coactivators. The absence of oxygen leads to the stabilization and translocation of HIF-α to the nucleus where it heterodimerizes with HIF-1β to form the HIF–α/1β complex. Then, this complex recruits transcriptional coactivator and regulates target gene expression. Histone lysine demethylases (KDMs) can directly sense oxygen to control cell fate by regulating the chromatin structure in a HIF-independent manner. For example, KDM6A and KDM5A are inactivated during hypoxia, causing hypermethylation of H3K27 (KDM6A target) and H3K4 (KDM5A target)

Studies have revealed that cellular responses to hypoxia are mediated by hypoxia-inducible factor (HIF)-dependent pathways and histone lysine demethylases (KDMs), as shown in Fig. 2. KDMs are oxygen-dependent enzymes that regulate histone methylation [65], which are novel oxygen sensors beyond HIF [66]. Certain histone demethylases, such as KDM6A and KDM5A, directly sense oxygen to regulate gene expression by controlling chromatin structure [66]. For example, hypoxia-induced KDM6A inactivation leads to the persistence of histone-3 lysine-27 trimethylation (H3K27me3), eventually blocking cellular differentiation [65].

With the ability to regulate the expression of hundreds of target genes, the HIF pathway plays a central role in coordinating cellular responses to oxygen deprivation. HIF consists of two distinct subunits: HIF-α (HIF-1α, HIF-2α, HIF-3α) and HIF-1β (also called ARNT, aryl hydrocarbon receptor nuclear translocator). Under hypoxic conditions, HIF-α proteins are stable and can be translocated into the nucleus, where they heterodimerize with HIF-1β proteins to form the functional HIF transcription factor complex. Following the recruitment of transcriptional coactivators, the HIF-α/HIF-1β complex regulates the expression of responsive genes by binding to the HRE located on the promoter regions of a large number of target genes [67]. In contrast, under normoxic conditions, HIF-α proteins are quickly degraded, failing to exert its functions [68].

Under normoxia, HIF-α is hydroxylated by prolyl hydroxylases (PHDs) and then recognized by E3-ubiquitin ligase von Hippel-Lindau (VHL), resulting in the rapid degradation of HIF-α protein [69, 70]. The activity of PHDs is related to Fe(II), 2-oxoglutarate (2OG), and oxygen. Under normoxia, dioxygen is delivered to the active site in the PHD2.Fe(II)0.2OG.HIF substrate complex through a single hydrophobic tunnel. This reversible binding of dioxygen is central to the hypoxia-sensing capacity of the PHDs, influencing the extent of HIF-α substrate prolyl hydroxylation [71]. The PHDs family has three canonical members: PHD1, PHD2, and PHD3. Each isoform has a differential function in regulating HIF-α activity [72]. In addition, studies in vitro have shown that the negative regulation of HIF-1α and HIF-2α by VHL is functionally distinct. Compared to HIF-2α, HIF-1α has a stronger affinity for VHL [73]. Additionally, different sites of proline hydroxylation play different roles in HIF-1α-pVHL interactions [74]. An additional oxygen-dependent hydroxylase involved in the regulation of the HIF pathway is the factor-inhibiting HIF (FIH). FIH is an asparagine hydroxylase that suppresses the transcriptional activity of HIF-α by preventing the recruitment of transcriptional coactivators without affecting the stability of HIF-α protein [75].

Notably, recent studies have revealed that the activation of HIF-α is not always correlated with hypoxia. In the study by Xiaotong Diao et al. [76], oleoylethanolamide (OEA) could selectively bind to the Per-ARNT-Sim-B (PAS-B) pocket of HIF-3α, resulting in the enhanced activity of HIF-3α. OEA is an oleic acid derivative that regulates food intake and metabolism. The identification of OEA provides evidence that endogenous small-molecule ligands can control the HIF pathways directly. It's interesting to note that some small-molecule compounds (described in detail below) can similarly bind to the PAS-B pocket, but they function as antagonists to suppress HIF-α activity, which may be related to their particular allosteric effects. In the study by Andrea L Casillas et al. [77], PIM1 kinase directly phosphorylated HIF-1α regardless of oxygen tension to prevent PHDs from binding to and hydroxylating HIF-1α, hence interrupting its degradation pathway. Dong Zhao et al. [78] discovered that the oncogene iASPP (inhibitor of apoptosis-simulating protein of p53) bound directly to VHL and prevented HIF-1α from degrading without affecting the PHD-mediated HIF-1α hydroxylation. In conclusion, the fact that the HIF pathway is regulated by a variety of cellular conditions highlights the importance of this pathway in numerous biological processes.

## Hypoxia-driven crosstalk between tumor cells and TAMs

**Fig. 3:**
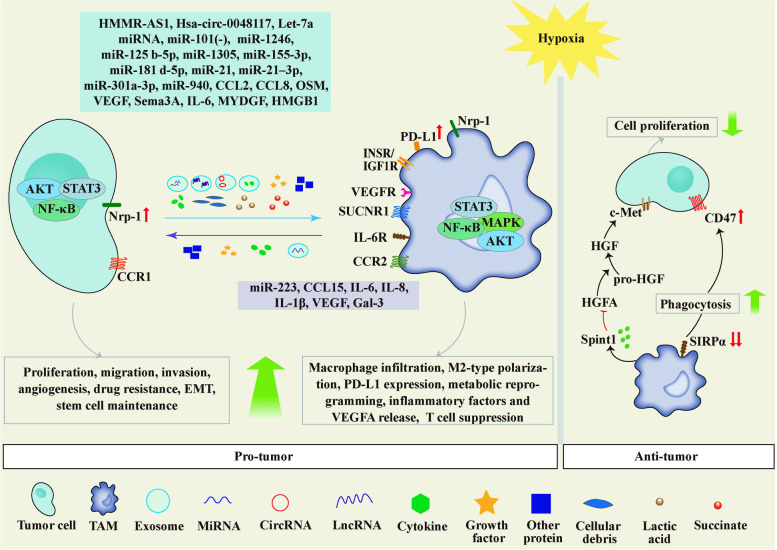
Hypoxia-driven crosstalk between tumor cells and tumor-associated macrophages (TAMs). The complex interplay between tumor cells and TAMs under hypoxia conditions may have tumor-promoting and tumor-suppressive consequences. The mediators that are responsible for tumor cell-to-TAM communication under hypoxia include exosomes, cytokines, growth factors, cellular debris, and oncometabolites. In addition, hypoxia can regulate the expression of cell surface ligands and receptors mediating cell signaling transduction

Extracellular vesicles, cytokines, growth factors, and proteins mediate reciprocal interactions between cells within TME to fulfill the growing demands of tumor cells [79]. The expression and release of these mediators are greatly affected by hypoxia. Thus, a comprehensive understanding of the mediators and pathways involved in the hypoxia-induced macrophage-cancer cell crosstalk should be helpful in finding accurate biomarkers and therapeutic targets. This section will summary the mediators and signaling pathways participating in macrophage-cancer cell crosstalk under hypoxia (Fig. 3) (Table 1).

**Table 1:** Recent studies on the mechanisms of hypoxia-driven crosstalk between tumor cells and TAMs

Donor cell	Receptor cell	Mediator	Mechanism	Effect	Ref
Non-small-cell lung carcinoma	TAMs	Exosomal miR-101	CDK8	Downregulated exosomal miR101 induces the secretion of IL1A and IL6 in macrophages and leads to inflammation in TME	[80]
Glioma	TAMs	Exosomal miR-1246	STAT3 and NF-κB	Promote M2-like macrophage polarization; promote tumor proliferation, migration and invasion	[81]
Multiple myeloma	TAMs	Exosomal miR-1305	Unknown	Promote M2-like macrophage polarization;	[82]
Epithelial ovarian cancer	TAMs	Exosomal miR-21–3p, miR-125 b-5p and miR-181 d-5p	SOCS4/5/STAT3	Promote M2-like macrophage polarization; promote tumor proliferation and migration	[83]
Endometrial cancer	TAMs	Exosomal miR-21	Unknown	Promote M2-like macrophage polarization	[84]
TAMs	epithelial ovarian cancer	Exosomal miR-223	PTEN-PI3K/AKT	Decrease apoptosis rate, increase cell viability, and enhance drug resistance	[85]
Pancreatic Cancer	TAMs	Exosomal miR-301a-3p	PTEN/PI3Kγ	Promote M2-like macrophage polarization; promote pancreatic cancer cells migration, invasion, and EMT	[86]
Glioma	TAMs	Exosomal IL-6 and miR-155-3p	IL-6-pSTAT3-miR-155-3p-autophagy-pSTAT3	Induce autophagy and M2-like polarization in macrophage; promote tumor proliferation and migration	[87]
Epithelial ovarian cancer	TAMs	Exosomal miR-940	Unknown	Promote M2-like macrophage polarization; promote tumor cell proliferation and migration	[88]
Esophageal Squamous Cell Carcinoma	TAMs	Exosomal Hsa-circ-0048117	miR-140/TLR4	Function as a ceRNA; promote M2-like macrophage polarization; promote tumor invasion and migration	[89]
Hepatocellular carcinoma	TAMs	Exosomal HMMR-AS1	miR-147a/ARID3A	Function as a ceRNA; promote M2-like macrophage polarization; promote tumor proliferation and growth	[90]
Melanoma	TAMs	Exosomal Let-7a miRNA	Insulin-Akt-mTOR	Recruit macrophages, promote M2-like polarization and enhance mitochondrial OXPHOS in macrophage	[91]
Non-small-cell lung cancer	TAMs	Exosome	HIF-1α	Upregulate PD-L1 in macrophage	[92]
Breast tumor	TAMs	OSM	mTORC2-Akt1	Promote M2-like macrophage polarization	[93]
TAMs	Gastric cancer	CXCL8 (IL-8)	CXCR1/2-JAK/STAT1	Deteriorate the GC malignant phenotype; promote IL-10 expression in tumor; IL-10 activates the macrophage IL-10/ NF-κB signaling pathway and induce M2 polarization	[94]
Cervical cancer	TAMs	CCL8	ZEB1–CCL8-CCR2–NF-κB	Enhance TAM migration	[95]
Lung cancer	TAMs	MCP-1 (CCL2)	NF-κB/HIF-1α	Recruit macrophages	[96]
Head and neck squamous cell carcinoma	TAMs	VEGF and IL-6	Unknown	Recruit and polarize macrophages	[97]
TAMs	Head and neck squamous cell carcinoma	CCL15	CCR1-NF-κB	Promote tumor tolerance to gefitinib	[97]
TAMs	Gastric Cancer	VEGF	PI3K-Akt and p38 MAP kinase	Promote the proliferation and invasion of cancer cell	[98]
Lewis lung carcinomas	TAMs	Sema3A	Nrp1/VEGFR1/PlexinA1/A4	Attract macrophages to the hypoxic region	[99]
Hepatocellular carcinoma	TAMs	MYDGF	Unknown	Enhance macrophage chemotaxis and inflammatory cytokines release, such as IL-6 and TNF-α; enhance self-renewal of cancer stem cells; promote tumor angiogenesis	[100]
TAMs	Breast cancer	Gal‐3	NF-κB via ROS	Promote tumor growth, metastasis, angiogenesis; enhance VEGFA secretion and glucose consumption in macrophage	[101]
Hepatocellular carcinoma	TAMs	HMGB1	Unknown	Exacerbate infiltration, M2-like polarization and IL-6 expression of macrophages; promote tumor EMT	[102]
TAMs	Breast tumor	Spint1	HIF-2α–Spint1/HGFA HGF/c-Met	Inhibit the proliferation of tumor cell	[103]
TAMs	Colon Cancer	SIRPα	SIRPα-CD47	Increase macrophage phagocytic activity	[104]
Cervical cancer	TAMs	Nrp-1	Unknown	Recruit and polarize macrophages towards the M2 phenotype	[105]
Hepatocellular carcinoma	TAMs	Cellular debris	TLR4/TRIF/NF-κB	Polarize macrophages into M2 type and promote the secretion of IL-1β	[106]
TAMs	Hepatocellular carcinoma	IL-1β	IL-1β/HIF-1α/COX-2	Enhance EMT of tumor cells	[106]
Lewis lung carcinoma and B16-F1 melanoma	TAMs	Lactic acid	HIF-1α	Induce the expression of VEGF and the M2-like polarization of TAMs	[107]
Lung Cancer	TAMs	Lactic acid	Unknown	Differentially affect TAM subset metabolism; trigger the T-cell suppressive capacity of TAMs	[108]
Lung Cancer	TAMs	Succinate	SUCNR1	Promote macrophage recruitment, migration and M2-skewed phenotype; M2 macrophages secret IL-6 to enhance cancer cell migration; induce cancer cell migration and EMT	[109]

### Exosomes

Exosomes are extracellular particles with diameters ranging from 40 to 160 nm that can be released into the cell's surrounding environment. Exosomes contain a variety of constituents, depending on their cellular origins, such as nucleic acids, proteins, lipids, amino acids, metabolites, and cytosolic [110–112]. Exosomes have an impact on tumor growth, metastasis, paraneoplastic syndromes and provide resistance to therapy, making them a research focus in the field of oncology [110]. Plenty of evidence suggests that hypoxic effects in the TME are mediated by exosomes that carry information in cell-to-cell communication [19]. Hypoxia exerts its effects on cancer-derived exosomes in many ways, including increasing exosome release [91], elevating exosomal heterogeneity (for example, size and cargo), and enhancing exosome target cell recognition and internalization [19]. The results of comprehensive proteomics [91] showed that exosomes secreted from hypoxic tumors contain elevated protein levels of: (1) chemokines, such as colony-stimulating factor 1 (CSF1), C–C motif chemokine 2 (CCL2), and endothelial monocyte-activating polypeptide 2 (EMAP2); (2) pro-tumorigenic molecules, including matrix metalloproteinases 2 (MMP2), procollagen-lysine, 2-oxoglutarate 5-dioxygenase 1 gene (PLOD1) and annexin A4 (ANXA4); (3) soluble inhibitory factors, like transforming growth factor beta (TGFβ), macrophage migration inhibitory factor (MIF) and ferritin heavy/light chain (FTH, FTL); (4) microRNAs processing proteins and growth factors, for example, argonaute 1 (AGO1), AGO3, hepatoma‑derived growth factor (HDGF). Some of these proteins, such as CSF1, CCL2, and EMAP2, can mediate macrophage recruitment and M2 polarization. The content of this section demonstrates that the key cargos in exosomes mediated the interaction between tumor cells and TAMs under hypoxia are microRNAs (miRNAs), followed by long noncoding RNAs (lncRNAs), circular RNA (circRNAs), and interleukins (ILs).

#### Exosomal MiRNAs and ILs

MiRNAs, a class of regulatory non-coding RNAs (ncRNAs), are frequently found in different exosomes and are involved in tumor proliferation, angiogenesis, metastasis and chemoresistance [113]. Tumor-derived exosomes generally contain one or more miRNAs, which are involved in different signaling pathways. The production of miRNAs in tumor-derived exosomes can be regulated by HIF-1α or HIF-2α [86].

MiR-1246 targets telomeric repeat binding factor 2 interacting protein (TERF2IP) and markedly promotes M2 macrophage polarization by activating the STAT3 pathway and inhibiting the NF-κB pathway, and ultimately leading to tumor proliferation, migration and invasion [81]. Moreover, miR-1246-rich exosomes derived from hypoxic tumor cells are delivered to normoxic tumor cells for inducing tumor migration and invasion [114]. TERF2IP (also known as RAP1), a member of the shelterin complex, plays a crucial part in protecting telomeric function and maintaining chromosome stability. It also acts as an essential modulator to enhance NF-kB signaling and attenuate STAT3 signaling [81, 115]. Of note, activating the NF-κB pathway and the STAT3 pathway can induce M1 and M2 gene expression, respectively [22, 116, 117]. In addition, NF-κB is be a crucial transcription factor that regulates the release of ILs from TAMs. For example, an in vitro study illustrated that macrophages transfected with NF-κB (p50) siRNA exhibited decreased expression levels of IL-10, VEGF, and matrix metalloproteinase-9 (MMP-9), whereas increased expression levels of IL-12, tumor necrosis factor-α (TNF-α), and IL-6 [118].

Exosomes released from hypoxic epithelial ovarian cancer cells deliver miR-21–3p, miR-125 b-5p and miR-181 d-5p to macrophages and induce M2 macrophage polarization, ultimately leading to tumor proliferation and migration [83]. Among these three miRNAs, miR-21–3p and miR-125 b-5p bind to SOCS4, whereas miR-21-3p and miR-181 d-5p bind to SOCS5, resulting in the decrease of SOCS4/5 expression and the increase of phosphorylated STAT3 [83]. To be clear, SOCS4 and SOCS5, members of suppressor of cytokine signaling (SOCS) families, are critical negative regulators of the JAK-STAT pathway.

Under hypoxia, total expression of let-7a miRNA (a well-known epigenetic tumor suppressor) in tumor cells is only about 30% of that in normoxia**,** whereas exosomal let-7a miRNA is increased by almost 25 times. These observations indicate that let-7a miRNA is extruded from tumor cells via exosomes [91]. Exosomal let-7a miRNA is transferred to TAMs and downregulates the expression of insulin-like growth factor 1 receptor (IGF1R), insulin receptor (INSR), insulin receptor substrate-1 (IRS-1) and IRS-2. The inhibition of insulin signaling-related genes can negatively regulate the insulin AKT-mTOR signaling pathway, leading to a metabolic shift from glycolysis to oxidative phosphorylation (OXPHOS) and eventually M2-like polarization in macrophages [91].

In addition, tumor-derived exosomal miRNAs can regulate inflammatory cytokine secretion in macrophages. Hypoxia has no influences in regulating IL1A or IL6 expression in macrophages, but instead dramatically promotes their expression in the co-culture with tumor cells. The reason for this could be that the tumor inhibitor miR101 is disturbed in tumor-derived exosomes under hypoxic stress, leading to the upregulation of cyclin-dependent kinase 8 (CDK8) in macrophages and the stimulation of IL1A and IL6 secretion in macrophages [80]. As a tumor inhibitor miRNA, miR101 is participating in various cancer-related biological processes by targeting multiple oncogenes. Thus, miR101 is considered to be a potentially novel approach for cancer therapy [119, 120]. CDK8 is an oncogene that functions as a transcriptional coactivator for several oncogenic transcription factors. [121]. IL1A and IL6 are two crucial inflammatory cytokines in macrophages, playing important roles in tumor development. IL-6 has multiple functions the activation of pro-oncogenic STAT3 signaling, the enhancement of cell motility, the reduction of cell–cell adhesion, the promotion of EMT, and the stimulation of cell proliferation [122]. Given that IL-1A has both pro- and anti-tumor effects, its role in cancer development is controversial. Results of in vivo experiments revealed that IL-1A overexpression in mice suppressed liver metastasis of lymphoma, which was related to an increase in CD8^+^ T-cells [123]. An in vivo and in vitro investigation on hepatocellular carcinoma (HCC) illustrated that tumor-derived IL-1A promoted tumor growth by increasing tumoral infiltration of myeloid-derived suppressor cells (MDSCs), which suppressed T and NK cell activation. In contrast, systemic administration of recombinant IL-1A protein exerted an anti-tumor effect by directly activating T cells. The location of released IL-1A is therefore crucial to understanding how it contributes to tumor growth [124]. Furthermore, the detailed function of IL-1A may be related to the type of cancer [125].

Hypoxic glioma-derived exosomes can induce macrophage autophagy and M2 polarization through their highly expressed IL-6 and miR-155-3p. IL-6 facilitates M2-like macrophage polarization either directly or indirectly through the IL-6-pSTAT3 pathway and the IL-6-autophagy-pSTAT3 pathway, respectively. Overexpression of miR-155-3p downregulates CREBRF gene expression and promotes autophagy in macrophages [87]. CREB3 regulatory factor (CREBRF), a negative regulator of CREB3 (cAMP responsive element binding protein 3), contributes an important part in hypoxia-induced autophagy in glioma cells [126]. Additionally, IL-6 can upregulate miR-155-3p expression by activating STAT3 in TAMs [87]. Autophagy plays a pivotal role in promoting M2-like macrophage polarization by activating the STAT3 pathway [127–129].

Tumor–derived exosomal miR-301a-3p, which is regulated by HIF-1α and HIF-2α, can be transferred to TAMs, promoting tumor cell EMT, migration, invasion, and metastatic potential. Exosomal miR-301a-3p mediates macrophages M2 polarization via downregulating PTEN (phosphatase and tensin homolog deleted on chromosome ten) expression and activating the PI3Kγ signaling pathway [86]. PTEN is a widely known tumor suppressor gene with phosphatase activity and regulates cell growth, proliferation, apoptosis, adhesion, migration, invasion, and genomic integrity [130]. PTEN can inhibit the PI3K/AKT pathway by dephosphorylation of phosphatidylinositol 3,4,5-trisphosphate (PIP3) [130]. It is worth noting that even a tiny decrease in PTEN levels can contribute to obvious cancer susceptibility and tumor progression [131]. Furthermore, miR-1305 [82], miR-21 [84], and miR-940 [88] in tumor-derived exosomes can promote M2 macrophage phenotype, but mechanisms of these miRNAs have not been thoroughly studied.

Under hypoxic conditions, macrophages can also secrete miRNAs via exosomes to regulate tumor biological functions. Exosomal miR223 derived from hypoxic TAMs is internalized into co-cultured tumor cells, resulting in the decreased apoptosis rate, increased cell viability, and enhanced drug resistance. Specifically, miR-223 down-regulates expression of PTEN and gradually increases PI3K/AKT signal activation [85]. However, inhibition of miR-223 expression cannot completely eliminate the promotion of chemoresistance by hypoxic macrophage-derived exosomes [85], indicating that the communication between tumor cells and macrophages under hypoxia is quite complex. Taken together, exosomal miRNA is a well-investigated mediator of tumor-macrophage communication under hypoxia.

#### Exosomal CircRNAs

CircRNAs are a novel class of nc-RNAs that have stronger stability than linear RNAs due to their covalently closed loops and have become a hotspot in recent years [132]. As a competitive endogenous RNA (ceRNA), circRNAs regulate gene expression through sponging miRNAs. For example, Hsa-circ-0048117 can be used as a ceRNA to inhibit the activity of miR-140. Tumor-derived exosomal Has-circ-0048117 inhibits miR-140 expression, upregulates the TLR4 expression, and promotes M2 polarization. Subsequently, Arg1, IL-10 and TGF-β secreted by M2 macrophages facilitate tumor cell invasion and migration [89]. TLR4 is a typical receptor from the Toll-like receptors (TLRs) family that is expressed on both immune cells and tumor cells, and its overexpression may lead to cancer progression [133, 134]. Paradoxically, TLR4 can cause macrophage polarization towards M1 [135]or M2 [136, 137].

#### Exosomal LncRNAs

LncRNAs are nc-RNAs that have more than 200 nucleotides and perform a variety of functions in the nucleus and cytoplasm. In the nucleus, they are involved in regulating chromosome architecture, modulating inter- and intrachromosomal interactions, remodeling chromatin, and directly regulating transcription. In cytoplasm, they can modulate mRNA stability, translation, and post-translation [138]. The newly discovered lncRNA hyaluronan-mediated motility receptor antisense RNA 1 (HMMR-AS1) [139] is found in tumor cytoplasm and is involved in cell proliferation, cell migration, and EMT [90]. Hypoxic condition can enhance the transcription of HMMR-AS1 by introducing the binding of promoter regions and HIF-1α. Tumor-derived exosomal HMMR-AS1 regulates M2-shifted polarization by miR-147a/A-T rich interacting domain 3a (ARID3A) axis [90]. Concretely, HMMR-AS1 functions as a ceRNA of miR-147a, limiting ARID3A degradation while increasing inhibition of M1 type polarization and promoting M2 type polarization [90].

#### Other exosomes

Exosomes released from intermittently hypoxic tumor cells also promote PD-L1 expression in macrophages, providing biological plausibility for explaining the underlying mechanisms of poor prognosis observed in patients with cancer and obstructive sleep apnea (OSA) [92]. Unfortunately, this study did not fully elucidate the specific exosome components that dominate this effect [92]. PD-L1 commonly expresses on TAMs in high grade serous ovarian cancers (HGSOC) at both the original and metastatic locations [140]. The binding of PD-L1 expressed on macrophages to PD-1 expressed on T-cells inhibits T cell cytotoxicity [141].

### Cytokines and growth factors

Cytokines, a class of diverse low-molecular weight proteins, include IL, colony-stimulating factors, chemokines, and tumor necrosis factors [142]. Chemokines can be categorized into four subclasses regarding to their amino acid motif at N-termini: CXC, CC, C, or CX3C, where C and X stand for cysteine and non-cysteine residues, respectively [143].

The inflammatory cytokine oncostatin M (OSM), which belongs to the IL-6 superfamily, is an essential part of the secretome of hypoxic cancer cells. OSM can enhance the expression of M2 macrophage surface markers (viz. CD206 and CD163) as well as functional markers (viz. arginase-1, and cyclooxygenase-2) in macrophages, which are involved in activation of mTOR signaling complex 2 (mTORC2) pathway [93]. Additional research showed that activated mTORC2 leads to M2 polarization by relaying signals through its effector kinases Akt, particularly Akt1, rather than PKCα. IL-4 is another classic mediator of M2 polarization by activating the mTORC2 pathway [93].

IL-8 (also known as CXCL8) is mainly produced by macrophages and plays a controversial role in regulating cancer progression [144]. Hypoxia increases IL-8 secretion significantly in macrophages but only slightly in gastric cancer (GC) cells [94]. Macrophage-derived CXCL8 induced by hypoxia can activate the JAK/STAT1 signaling pathway through binding to CXCR1/2 expressed on GC cells, leading to GC invasion and proliferation. The activation of STAT1 directly upregulates the expression of IL-10, stimulating M2 polarization of macrophages through the NF-κB signaling pathway. CXCL8 production is further encouraged by NF-κB activation [94].

The expression level of C–C chemokine ligand 8 (CCL8) is increased by zinc finger E-box binding homeobox 1 (Zeb1) via directly interacting with the CCL8 promoter in the hypoxic TME. Subsequently, CCL8 promotes TAMs infiltration via CCR2–NF-κB pathway, and this result is typically associated with a poor prognosis in cervical cancer [95]. Zeb1 is a typical transcription factor that is widely expressed in carcinomas and has a significant role in the development of cancer by promoting EMT and chemoresistance in cancer cells [145]. Monocyte chemoattractant protein-1 (MCP-1), also known as CCL2, is a key chemokine controlling macrophage migration and invasion [146]. Under hypoxic stress, NF-κB/HIF-1α activation encourages lung cancer cells to secrete MCP-1, which furthers the accumulation of macrophages [96].

Under a hypoxic microenvironment, vascular endothelial growth factor (VEGF) and IL-6, generated by head and neck squamous cell carcinoma (HNSCC) attract macrophages and polarize them to the M2 type. Later, M2-type TAMs release CCL15 through the HIF-2α pathway, which leads to gefitinib resistance in HNSCC via CCL15-CCR1-NF-κB pathway [97]. Moreover, VEGF can also be secreted from macrophages, and both its mRNA and protein levels rise in response to hypoxia in a time-dependent manner. The upregulation of VEGF increases the phosphorylation of Akt and p38, contributing to the proliferation and invasion of tumor cells [98]. Under hypoxic conditions, TAMs-derived VEGF binds to its receptor VEGFR on tumor cells, activating the PI3K-Akt and p38 MAP kinase pathways to promote tumor cell proliferation and invasion [98].

Semaphorin3A (Sema3A) is a membrane-bound protein that has been shown to be a prognostic marker for patients with metastatic colorectal cancer (mCRC) [147]. In vivo studies have revealed that Sema3A is an endogenous inhibitor of angiogenesis that counteracts angiogenic factors like VEGF-A. Sema3A has the ability to regulate tumor blood vessels, alleviate tumor hypoxia, and inhibit tumor growth [148]. Sema3A expression is higher in hypoxic tumor single cell suspensions than in normoxic conditions [99]. In vivo and in vitro studies have documented that Sema3A drives TAMs toward hypoxic niches via the Sema3A–neuropilin-1 (Nrp1) pathway. Following macrophage localization in the hypoxic environment, Nrp1 is downregulated, and Sema3A captures TAMs locally via Nrp1-independent plexinA1-plexinA4-mediated stop signals [99]. Finally, hypoxic TAMs acquire protumoral phenotypes. The absence of Sema3A leads to a more M1-like phenotype and a reduced tumor growth [99]. Nrp-1 is a pleiotropic single-pass transmembrane protein that functions as a co-receptor to many extracellular ligands [149]. In vitro study showed that the expression of Nrp-1 in tumor cells was upregulated under hypoxic situation, which resulted in recruiting more macrophages and educating them into M2-phenotype [105]. However, more research is required to fully understand how Nrp-1, which is produced by cancer cells, promotes M2 macrophage polarization under hypoxic conditions.

Myeloid-derived growth factor (MYDGF), which is generated when tissue is damaged, exerts a crucial role in regulating neutrophil interstitial motility and inflammation in a way that is HIF-1α dependent [150]. Hepatocellular carcinoma contains hypoxia-induced MYDGF in its cytoplasm and cell membrane, which can stimulate tumor angiogenesis and boost the potential of cancer stem cells to self-renew. Additionally, tumor-derived MYDGF promotes the release of inflammatory cytokines including TNF-α and IL-6 and increases macrophage infiltration, all of which ultimately aid in the growth of tumors [100]. However, the molecular mechanism of MYDGF action in tumor progression is still unclear, and it merits further research.

### Binding proteins and protease inhibitors

Galectin-3 (Gal‐3), a member of the β-galactoside binding protein family [151], is both a prognostic indicator and a potential target for cancer treatment [152–154]. Gal‐3 is also expressed in tumor tissues in a HIF-1α-dependent manner [155], causing an increase in PD‐L1 level via STAT3 phosphorylation in carcinomas [156]. Gal‐3 secreted by TAMs during hypoxia promotes tumor metastasis and angiogenesis, which is highly dependent on the degree and duration of hypoxia [101]. Although the expression level of HIF-1α is elevated in hypoxic TAMs, HIF-1α inhibitors have no effect on the expression of Gal-3 there, suggesting that HIF-1α may not be involved in Gal-3 expression in hypoxic TAMs. Interestingly, HIF-1α inhibitor 2ME2 can upregulate Gal-3 expression in normoxia but not in hypoxia. Further research revealed that the upregulation of Gal‐3 expression in hypoxic TAMs is associated with an increase in intracellular reactive oxygen species (ROS) level via activation of NF-κB nucleation. In addition, Gal-3 overexpression enhances VEGFA secretion and glucose consumption in TAMs [101].

As a nuclear DNA-binding protein, high-mobility group box 1 (HMGB1) can be robustly upregulated via HIF-1α signaling after prolonged exposure to hypoxia in tumors. Increased HMGB1 levels encourage macrophage infiltration and cytokine expression(i.e. IL-6). Subsequently, macrophage-derived IL-6 activates STAT3 signaling and promotes EMT in tumor cells [102].

However, other studies noticed that tumor-infiltrating macrophages in the hypoxic microenvironment may have tumor suppressing effects. Under hypoxic conditions, HIF-2α highly expressed in TAMs induces the secretion of the serine protease inhibitor Spint1. Spint1 is then released into TME to block the serine protease HGF activator (HGFA), preventing the cleavage of pro-HGF into active hepatocyte growth factor (HGF) [103]. When activated HGF binds to the c-Met receptor on tumor cells, it activates several signaling pathways, including MAPK, PI3K/AKT, and STAT3, which promotes tumor growth and therapeutic resistance [157]. Thus, TAM-secreted Spint1 can reduce tumor cell proliferation.

### Ligand-receptor interaction between tumor cells and TAMs

The binding of CD47 (cluster of differentiation 47) on tumor cells to SIRPα (signal-regulatory protein α) ligand on macrophages is a typical tumor escape mechanism. The "don't eat me" signal is released by the CD47 receptor when it binds to SIRPα, which impairs the phagocytic activity of macrophages [158]. HIF has been shown to activate highly expressed CD47 in various cancer types [158]. However, hypoxia may have beneficial effects on cancer therapy via SIRPα-CD47 axis. Colon cancer has a better prognosis than other cancer types because it has higher levels of macrophage infiltration and HIF-1α expression [104]. Hypoxia can decrease SIRPα expression in macrophages while simultaneously increasing CD47 expression in colon cancer cells. The heightened signal of "don't eat me" is countered by the reduced SIRP expression level, increasing the phagocytic capacity of macrophages [104]. Therefore, HIF-1α does have the ability to enhance phagocytosis of macrophages, which may be dependent on cancer types [158].

### Tumor cell debris

Tumor necrotic debris caused by hypoxia can release different signals leading to cancer progression [159]. Macrophages can accumulate in perivascular and perinecrotic niches in tumors [160] where they can operate as immune scavengers to sweep away cellular debris [161]. An interesting study demonstrated that the necrotic debris from severely hypoxic cancer cells modulates the communication between tumors and TAMs [106]. Under conditions of moderate hypoxia, HIF-1α facilitates IL-1β secretion in macrophages. When exposed to severe hypoxia, necrotic cancer cell debris can stimulate IL-1β secretion in macrophages via TLR4/TRIF/NF-κB signaling. Specifically, necrotic debris enhances TLR4 signaling by attracting more TLR4 receptors to the macrophage membrane and activating TIR domain-containing adapter-inducing interferon-β (TRIF). Following then, phosphorylated NF-κB is up-regulated, resulting in macrophage M2 polarization and IL-1β secretion. Macrophage-derived IL-1β activates the IL-1β/HIF-1α/COX-2 axis, enhancing tumor cell EMT and promoting tumor invasion and metastasis [106].

It is worth noting that IL-1β in the TME can be an important driver of immune suppression. For instance, in mouse models of spontaneous breast cancer metastasis, IL1β stimulates IL17 expression from γδ T cells, leading to neutrophil accumulation via systemic induction of G-CSF. Neutrophils inhibit CD8^+^ T cell activation, allowing cancer cells to spread [162]. Interestingly, the results of IL1β mRNA expression in diverse cell populations separated from the transplanted tumors indicate that macrophages are the most abundant IL1β-expressing cell type [162]. These findings also highlight the importance of cross-talk between immune cells in influencing immune responses in tumors. Similarly, Máté Kiss et al. [163] also observed that IL1β exerted an immune-suppressive function in TME in two distinct mouse models. The researchers found that increased IL1β production within tumors was released mainly by neutrophils, monocytes, and macrophages. A noteworthy finding in that study was that the immunostimulatory major histocompatibility complex (MHC)-II^high^ TAMs produced large amounts of IL1β.

The NF-κB pathway has been identified as a critical regulator of macrophage behavior in the TME. Interestingly, NF-κB pathway exerts dual effects on macrophage polarization—both promotion and inhibition of M1 polarization. Studies in vitro showed that transfection of NF-κB (p50) siRNA into M2-like macrophages leaded to the anti-tumorigenic M1 phenotype [118]. Similarly, an in vivo study suggested that specific blockade of NF‐κB signaling in macrophages could switch macrophages from a M2 to a M1 phenotype [164]. However, some studies in vivo showed that the increased NF-κB activity in macrophages resulted in reduced tumor burden and persistent macrophage M1 polarization [165]. There are five members of the NF-κB family of transcription factors: p65 (RELA), p50 (NFKB1), p52 (NFKB2), c-REL, and RELB [166]. These members can couple to form different homo- or heterodimers, which have opposing effects on macrophage polarization, depending on the source of macrophage populations and the way that macrophages are activated [167]. Lipopolysaccharide, for example, promotes the overexpression of p50-p50 homodimers, allowing M1 to M2 macrophage reprogramming [168–170]. In contrast, Bufalin promotes the overexpression of p65-p50 heterodimers, leading to the transition of macrophage from M2 to M1 [171]. Therefore, in order to better understand the multifaceted role that NF-κB plays in regulating TAMs function, it may be useful to investigate the exact functions of various NF-κB dimers.

### Oncometabolites

Under hypoxic conditions, tumor cells may undergo metabolic reprogramming allowing them to shift from oxidative phosphorylation to anaerobic glycolysis. Succinate, an intermediate of the tricarboxylic acid (TCA) cycle, and lactate, an end product of glycolysis, are two examples of tumor metabolites produced by hypoxic tumor cells that can influence macrophage activity [172].

Lactic acid shuttles among different cells within the TME, acting not only as a stromal cell energy supplier, but also as a signaling molecule to intensify crosstalk between tumor cells and adjacent cells [173]. In vivo and in vitro study showed that lactate induces VEGF expression and M2-like polarization of TAMs, both of which are mediated by HIF-1α [107]. Lactate targets the protein-coupled receptors on the surface of the TAMs membrane and induces M2-type polarization via the PKA/CREB pathway [174]. Additionally lactic acid produced under hypoxia is believed to be a weapon for activating pro-angiogenic TAMs and increasing PD-L1 protein expression in TAMs [172, 175, 176]. It's important to note that in vitro research indicated that lactate differentially influenced TAM subgroup metabolism [108]. These subsets are known to reside in different intratumoral locations, with MHC-II^lo^ TAMs being enriched in hypoxic tumor areas. Lactate promotes oxidative metabolism in MHC-II^lo^ TAMs while inhibiting it in MHC-II^hi^ TAMs. Furthermore, in the presence of lactate, MHC-II^lo^ TAMs showed an improved ability to inhibit T cells.

Extensive evidences showed a positive synergistic relationship between hypoxia and lactate [177]. When normoxic and hypoxic macrophages are treated with different lactate doses, the protein levels of ARG1 increase concomitantly in hypoxic macrophages but not in normoxic ones, indicating that the combination of low oxygen and lactate is already sufficient enough to trigger Arg-1 expression [177]. Moreover, a high concentration of lactic acid causes medium acidification, which kills macrophages rather than causing Arg-1 expression. Macrophages have the ability to detect the presence of hypoxia and lactate. These signals can then be integrated with phenotypic responses by MAPK signaling, which results in the release of pro-angiogenic cytokines like VEGFA [177]. In-depth bioinformatics analysis of macrophages transcriptome data indicated that lactate has only mild impacts on macrophages under normoxic conditions. When lactate is combined with hypoxia, macrophages become significantly more M2-polarized via the HIF-1, Hedgehog and mTOR pathways is observed [178]. However, it is important to note that most of the studies on the effect of lactate on macrophages were conducted under normoxic conditions, rather than hypoxic conditions. The information in this section may serve as a reminder of the importance of conducting research under hypoxic conditions.

Succinate is a typical TCA cycle intermediate that promotes inflammation and can accumulate in macrophages in response to lipopolysaccharide (LPS). Succinate accumulation robustly boosts HIF-1α protein levels, leading to increased secretion of IL-1β from macrophages [179]. Specifically, succinate produced by tumors is released into extracellular milieu and interacts to succinate receptor (SUCNR1) on the membrane of macrophages. As a result of this binding, the PI3K-HIF-1 axis is activated, which causes macrophage recruitment, migration, and an M2-skewed phenotype. M2 polarized macrophages secret IL-6 to enhance cancer cell migration. Meanwhile, tumor-derived succinate also activates SUCNR1 on the membrane of tumor cells to induce cancer cell migration and EMT through the PI3K/HIF-1α pathway [109]. These findings demonstrated that the tumor-derived succinate has great potential to be a novel target for anti-tumor therapy because of its ability to control TAM polarization and tumorigenic pathway.

## HIF-1α/2α inhibitors for cancer treatment in clinical studies

**Fig. 4:**
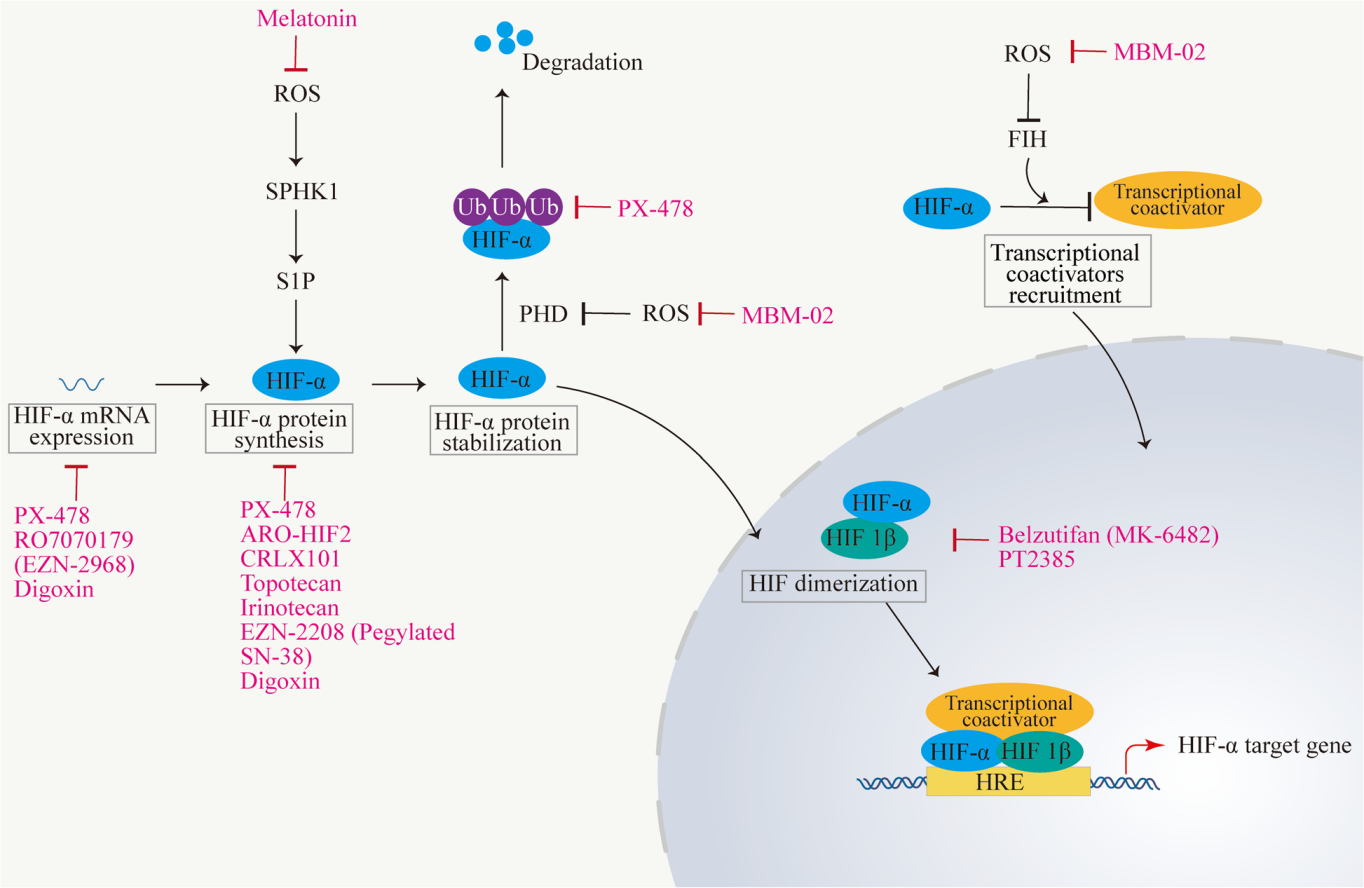
Mechanisms of action of the HIF inhibitors currently on the market or under clinical trials. HIF inhibitors target HIF on different levels, ranking from transcription, translation, protein stabilization, transcriptional coactivators recruitment, and dimerization. The clearance of ROS leads to decreased HIF-α stabilization and accumulation

As previously mentioned, hypoxia not only affects the biological function of tumor cells and macrophages but also the communication between them, triggering a series of signal pathways to support tumor survival. The activation of HIF-1α/2α is one of the main initiators of macrophage-cancer cell interaction. Inhibiting the activity of HIF-1α/2α can therefore open up new possibilities for tumor treatment approaches. This section will provide insights for therapeutic development by summarizing those pharmaceuticals that successfully entered clinical trials or the market for inhibiting HIF (Fig. 4) (Table 2).

**Table 2:** Clinical trials of HIF Inhibitors in cancers (https://clinicaltrials.gov)

Drug name	Drug type	Target	Condition or disease	Trial identifier	Phase
RO7070179 (EZN-2968)	Nucleic acid drug	HIF-1α	Hepatocellular Carcinoma	NCT02564614	I
			Advanced Solid Tumors With Liver Metastases	NCT01120288	I
			Advanced Solid Tumors or Lymphoma	NCT00466583	I
EZN-2208 (Pegylated SN-38)	Drug repurposing	HIF-1α	Neoplasms	NCT01251926	I
			Advanced Solid Tumors Lymphoma	NCT00520637 NCT00520390	I
PX-478	Small-molecule drug	HIF-1α	Advanced Solid Tumors or Lymphoma	NCT00522652	I
Melatonin	Drug repurposing	HIF-1α	Locally Advanced Oral Squamous Cell Carcinoma	NCT04137627	III
CRLX101	Drug repurposing	HIF-1α	Ovarian Cancer Fallopian Tube Cancer Primary Peritoneal Cancer	NCT01652079	II
Topotecan	Drug repurposing	HIF-1α	Neoplasms	NCT00117013	I
Irinotecan	Drug repurposing	HIF-1α	Refractory Solid Tumors in Children	NCT01282697	I
Digoxin	Drug repurposing	HIF-1α	Breast Cancer	NCT01763931	II
ARO-HIF2	Nucleic acid drug	HIF-2α	Advanced Clear Cell Renal Cell Carcinoma	NCT04169711	I
NKT2152	Small-molecule drug	HIF-2α	Advanced Clear Cell Renal Cell Carcinoma	NCT05119335	I/II
PT2385	Small-molecule drug	HIF-2α	Recurrent Glioblastoma	NCT03216499	II
			VHL Disease-Associated Clear Cell Renal Cell Carcinoma	NCT03108066	II
			Advanced Clear Cell Renal Cell Carcinoma	NCT02293980	I
			Renal Cell Carcinoma	NCT04989959	I
DFF332	Small-molecule drug	HIF-2α	Advanced/Relapsed Renal Cancer & Other Malignancies	NCT04895748	I
Belzutifan	Small-molecule drug	HIF-2α	Pheochromocytoma/Paraganglioma Pancreatic Neuroendocrine Tumor	NCT04924075	II
			Carcinoma, Renal Cell	NCT04846920	I
MBM-02	Small-molecule drug	HIF	Glioblastoma Multiforme	NCT04874506	II
			Prostate Cancer Recurrent Biochemical Recurrent Prostate Cancer	NCT04876755	II

### Small-molecule inhibitors

The PAS-B binding pocket on the HIF-2α contains a unique hydrophilic cavity that can accommodate a small molecule, which may result in a conformation change in HIF-2α and disruption of its interaction with ARNT [180, 181]. Accordingly, HIF-2α small-molecule inhibitors are discovered, such as Belzutifan (Welireg™, MK-6482) [182] and PT2385 [183]. Despite the high sequence identity between HIF-2α and HIF-1α, these small-molecule inhibitors are highly selective in dissociating the HIF-2α/ARNT heterodimer while having no effect on HIF-1 function [181, 184].

Belzutifan is the first FDA-approved treatment for Von Hippel-Lindau (VHL) disease in patients with renal cell carcinoma (RCC), central nervous system (CNS) hemangioblastomas, or pancreatic neuroendocrine tumors (pNET) without the request of immediate surgery [185–187]. VHL disease is a rare autosomal dominantly inherited tumor syndrome caused by germline mutation or deletion of VHL gene [188, 189]. The incidence of RCC patients with VHL disease is high due to VHL gene inactivation and constitutive activation of the transcription factor HIF-2α [190]. Belzutifan is also expected to be used in the treatment of polycythemia and multiple paragangliomas (the Pacak–Zhuang syndrome), which are caused by somatic mosaicism for an activating mutation in EPAS1 [191]. Several ongoing clinical trials are currently focusing on the evaluation of the efficacy of belzutifan in combination with other medicines, such as pembrolizumab, Lenvatinib, and cabozantinib (ClinicalTrials.gov Identifier: NCT04976634, NCT05239728, NCT03634540, NCT04736706, NCT05030506, NCT04626518, NCT04586231, NCT04626479). There are also some clinical trials of belzutifan used alone for other tumors (Table 2). It is hoped that these studies will yield excellent patient survival data.

PT2385, a small molecule drug, is the first HIF-2α antagonist progressed into clinical trials [192]. PT2385 can inhibit HIF-2 dimerization in healthy tissue and ccRCC metastases [193, 194]. PT2385 can significantly alleviate the undesirable adverse effects of sorafenib through inhibiting HIF-2α, increasing androgen receptor (AR) and suppressing downstream pSTAT3/pAKT/pERK pathways [195].

PX-478 is an active HIF-1α small-molecule inhibitor with potent antitumor activities [196, 197]. PX-478 can inhibit HIF-1α protein levels, transactivating activity, and deubiquitination. In addition, PX-478 prevents the synthesis of VEGF that is generated by hypoxia in various cancer cell lines [198]. By inhibiting the HIF-1α/lysyl oxidase-like 2 (LOXL2) signaling pathway, PX-478 can enhance immunotherapeutic effectiveness and reduce the EMT phenotypes induced by hypoxia [199]. PX-478 can drastically reduce the expression level of granulocyte–macrophage-colony-stimulating factors (GM-CSF) and the incidence of perineural invasion (PNI) in pancreatic ductal adenocarcinoma (PDAC) [200].

Cycling hypoxia increases the production of ROS, which promotes HIF-1α and NF-κB activation in tumor cells [201]. ROS is an important mediator of HIF-stability by inhibiting the activity of PHD and FIH in the cytoplasm [201]. MBM-02, also known as Tempol, is a dual-specific HIF-1 and HIF-2 inhibitor (ClinicalTrials.gov Identifier: NCT04876755). As a well-known antioxidant, MBM-02 promotes the clearance of ROS and inhibits cycling hypoxia-induced chemoresistance [202]. In addition, DFF332 (ClinicalTrials.gov Identifier: NCT04895748) and NKT2152 (ClinicalTrials.gov Identifier: NCT05119335) are also small molecules that inhibit HIF2α.

### Nucleic acid therapeutics

Due to their high target-specificity, nucleic acid therapeutics, such as miRNA-based molecules, lncRNAs, small interfering RNAs (siRNAs), antisense oligonucleotides (ASOs), mRNA therapeutics, and nucleic acid aptamers, have recently been successful in emerging into a highly attractive class of medicines [203].

RO7070179 (EZN-2968) is an ASO specifically targeting HIF-1α in a synthetic locked nucleic acid (LNA) form, which can reduce HIF-1α mRNA levels [204]. ASOs typically contain < 20 mER DNA or RNA nucleotides and can target mRNAs that are largely degraded through RNAse H-mediated cleavage. ASOs also inhibit the interaction between its targeted mRNAs and their paired enzymes, which blocks the transcription or translation of target genes [205, 206]. LNA-based oligonucleotides offer the advantages of remarkable stability, low off-target events, and high target-mRNA binding affinity [207, 208].

ARO-HIF2 is a synthetic double-stranded RNA interference (RNAi) trigger with an αvβ3 targeting ligand designed to silence HIF-2α expression. RNAi is a natural protective mechanism induced by double stranded RNAs (dsRNAs), leading to efficient and specific degradation of homologous mRNA [209]. Integrins αvβ3, which is related to tumor progression and metastasis, is frequently overexpressed in ccRCC and can be selectively bound by ARO-HIF2 [210]. The results of the phase I clinical trial provides preliminary evidence for the safety and efficacy of ARO-HIF2 in patients with advanced ccRCC [211].

### Drug repurposing

Compared with traditional de novo drug application, drug repurposing has become an effective alternative drug therapeutic strategy due to its lower risk of failure, reduce costs, and higher efficiency [212]. As shown in Table 2, there are some examples of drug repurposing to target hypoxia signaling in cancer.

Camptothecin (CPT) and its analogs (including SN-38, topotecan, and irinotecan) are important Topoisomerase I inhibitors that can block HIF-1α expression [213]. CPT has been approved for its ability to decrease the number of cancer stem cells (CSCs) and inhibit the accumulation of HIF-1α [214]. CPT can initiate the transcription of long noncoding antisense RNAs at the 5′ and 3′ ends of the HIF-1α, leading to posttranscriptional regulation of gene expression. Interestingly, low camptothecin concentrations have an effect on miR expression profiles, particularly increasing miR-17-5p and miR-155, which are two important players in reducing of HIF-1α protein accumulation and activity [215]. CRLX101 (NLG207) is a nanoparticle-drug conjugate (NDC) of CPT designed to overcome the poor physicochemical properties of CPT and allow more of CPT to accumulate at tumor sites. CRLX101 plus enzalutamide has been shown to be effective in preclinical prostate cancer models with enzalutamide resistance, and clinical trials are currently underway (NCT03531827) [216]. SN-38, the active metabolite of Irinotecan (CPT-11), can overcome hypoxia-induced chemoresistance [217] and inhibit the radiation-induced up-regulation of HIF-1α [218].

Additional data suggested that melatonin may also be a potent anti-tumor agent, inhibiting hypoxia-mediated tumor survival, angiogenesis, invasion, and migration [219]. Melatonin can block tumor angiogenesis by reducing HIF-1α protein expression in tumors [220]. Specifically, melatonin can inhibit the sphingosine kinase 1 (SPHK1) signaling pathway and impairs ROS generation in hypoxic cancer cells [221]. Under hypoxia, SPHK1 is activated by ROS to promote the accumulation of HIF-1α and initiate its transcriptional activity [221, 222]. Digoxin is also able to inhibit HIF-1α protein translation and HIF-2α mRNA expression, leading to anti- tumor effects [223].

## Conclusion and future perspective

Hypoxia is a critical factor that affects the communication between tumor cells and TMAs. Hypoxia influences the crosstalk between TAMs and tumor cells via vast multifunctional exosomes, cytokines, growth factors, cellular debris, oncometabolites, and a variety of ligands and receptors on the cell surface. Hypoxia-induced interaction between tumor cells and TAMs promotes tumor proliferation, migration, invasion, angiogenesis, drug resistance, EMT, and cancer stem cell self-renewal. In addition, hypoxia also promotes macrophage phagocytosis, which inhibits tumor cell proliferation. Therefore, hypoxia is a double-edged sword and a non-negligible factor in anti-tumor treatment, which needs further research to evaluate the evidences.

As Semenza stated in 2017, "It is ironic that hypoxic cancer cells… would be the last to capture the attention of oncologists." [224]. The majority of studies are still performed under normoxia, frequently ignoring the importance of hypoxia. As a result, more emphasis should be placed on hypoxic exploration. Oxygen level and duration have an impact on the stabilization of HIF-1α and HIF-2α [225], which in turn activates different signal pathways. Thus, more attention need to be paid on the detailed hypoxia level [61]. Plenty of studies have proved that HIF pathway is the core mechanism of cell hypoxic adaptation. However, the observation of oxygen sensing by KDMs suggested that additional oxygen sensors independent of HIF may be presented. The mechanisms of tumor hypoxic adaptation are more complex than currently envisaged. In clinical studies, HIF inhibitors are currently divided into three categories: small molecule drugs, nucleic acid drugs and drug repurposing. Applying strategies to suppress the negative effects of hypoxia appears to be useful to overcome malignant tumors, especially an already-approved HIF-2α inhibitor that has shown promising therapeutic activity, which has greatly enhanced our confidence in developing HIF inhibitors. In the future, we anticipate HIF pathway inhibitors being a cornerstone of cancer treatment.

## Data Availability

Not applicable.

## Abbreviations

TAMs: Tumor-associated macrophages
TME: Tumor microenvironment
EMT: Epithelial mesenchymal transition
HbCO: Carboxyhemoglobin
IH: Intermittent hypoxia
H-R cycles: Cycles of hypoxia and reoxygenation
HRE: Hypoxia response element
HIF-1: Hypoxia-inducible factor 1
ARNT: Aryl hydrocarbon receptor nuclear translocator
PHDs: Prolyl hydroxylases
FIH: Factor inhibiting HIF
VHL: Von Hippel-Lindau
KDMs: Histone lysine demethylases
PIM: Proviral insertion site in Moloney murine leukaemia virus
miRNAs: MicroRNAs
lncRNAs: Long noncoding RNAs
circRNAs: Circular RNA
IL: Interleukin
ncRNAs: Non-coding RNAs
TERF2IP: Telomeric repeat binding factor 2 interacting protein
SOCS: Suppressor of cytokine signaling
IGF1R: Insulin-like growth factor 1 receptor
INSR: Insulin receptor
IRS: Insulin receptor substrate
OXPHOS: Oxidative phosphorylation
CDK8: Cyclin-dependent kinase 8
CREB3: CAMP responsive element binding protein 3
PTEN: Phosphatase and tensin homolog deleted on chromosome ten
PIP3: Phosphatidylinositol 3,4,5-trisphosphate
ceRNA: Competitive endogenous RNA
TLRs: Toll-like receptors
HMMR-AS1: Hyaluronan-mediated motility receptor antisense RNA 1
ARID3A: A-T rich interacting domain 3a
OSA: Obstructive sleep apnea
OSM: Oncostatin M
mTORC2: MTOR signaling complex 2
CCL8: C–C chemokine ligand 8
Zeb1: Zinc finger E-box binding homeobox 1
MCP-1: Monocyte chemoattractant protein-1
VEGF: Vascular endothelial growth factor
MYDGF: Myeloid-derived growth factor
Gal‐3: Galectin-3
ROS: Reactive oxygen species
HMGB1: High-mobility group box 1
HGF: Hepatocyte growth factor
CD47: Cluster of differentiation 47
SIRPα: Signal-regulatory protein α
Nrp-1: Neuropilin-1
TRIF: TIR domain-containing adapter-inducing interferon-β
TCA: Tricarboxylic acid
HK2: Hexokinase2
PFKP: Phosphofructokinase, platelet
PKM2: Pyruvate kinase M2
SUCNR1: Succinate receptor
AR: Androgen receptor
LOXL2: Lysyl oxidase-like 2
GM-CSF: Granulocyte–macrophage-colony-stimulating factors
siRNAs: Small interfering RNAs
ASOs: Antisense oligonucleotides
LNA: Locked nucleic acid
RNAi: RNA interference
dsRNAs: Double-stranded RNAs
CPT: Camptothecin
CSCs: Cancer stem cells
NDC: Nanoparticle-drug conjugate
SPHK1: Sphingosine kinase 1
S1P: Sphingosine 1-phosphate
